# A mass spectrometric method for quantification of tryptophan-derived uremic solutes in human serum

**DOI:** 10.14440/jbm.2017.182

**Published:** 2017-07-31

**Authors:** Anqi Zhang, Keshab Rijal, Seng Kah Ng, Katya Ravid, Vipul Chitalia

**Affiliations:** 1 Metabolomics Core, Department of Medicine, Boston University School of Medicine, Boston, MA, USA; 2 Renal Section, Department of Medicine, Boston University School of Medicine, Boston, MA, USA; 3 Whitaker Cardiovascular Institute and the Department of Medicine, Boston University School of Medicine, Boston, MA, USA

**Keywords:** uremic solutes, mass spectrometry, indoxyl sulfate, kynurenine

## Abstract

In addition to various physiologic roles, emerging evidence strongly points to pathogenic roles of tryptophan and of its metabolites, especially in diseases such as renal failure. Accurate estimation of levels of these metabolites in blood is important to mechanistically probe their contribution to disease pathogenesis, while clinically, such a panel can be used to risk stratify patients for a clinical phenotype. Herein, we describe a comprehensive liquid chromatography-mass spectrometry (LC/MS)-based method to determine the level of tryptophan and its metabolites (kynurenine, kynurenic acid, xanthurenic acid, anthranilic acid, indoxyl sulfate and indoxyl acetate). Human sera samples were processed through a C18 column followed by application of a binary gradient and quantitation by MS/MS. The linearity, lower limit of detection, inter- and intraassay variabilities and recovery were determined, yielding a precise, reproducible method for all the metabolites. Unlike previous studies, we further validated these methods in a well-characterized set of human sera from end stage renal disease patients compared to age-, gender- and ethnic-background matched human controls. Overall, we report an optimized LC/MS-based estimation of a comprehensive panel of tryptophan-derived metabolites with quality features within FDA standards, underscoring their readiness for translational use.

## INTRODUCTION

**Figure 1.**
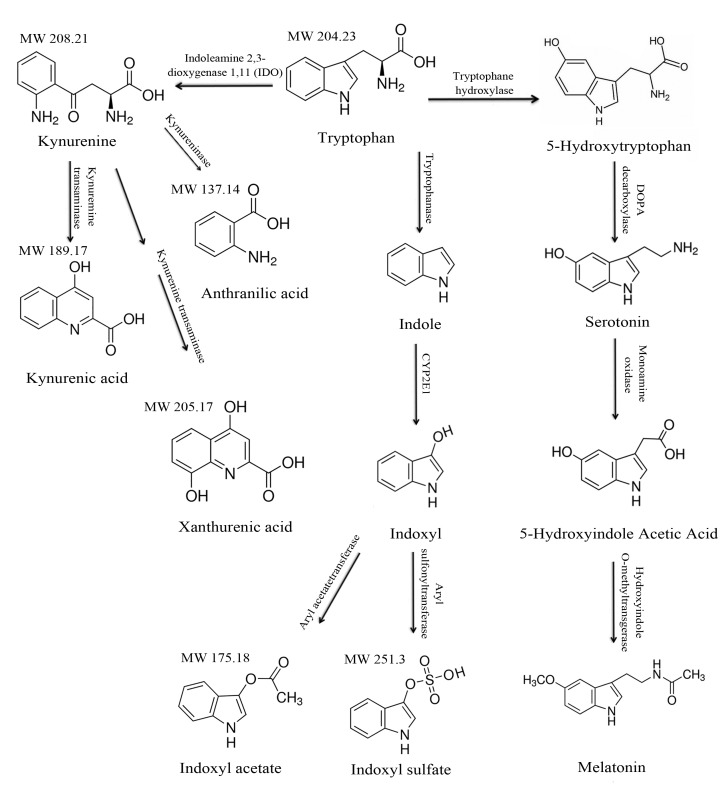
Tryptophan metabolism and the structures of its metabolites. The major metabolic pathways of tryptophan are shown. Tryptophan undergoes metabolism through the kynurenine and methoxyindole pathways. Tryptophan also undergoes fermentation by colonic bacteria, resulting in indol metabolites, which is the major source of uremic toxins.

Tryptophan is an essential aromatic amino acid that undergoes extensive metabolism in different organs and cells resulting in an array of products through several intermediates [1]. Tryptophan undergoes metabolism through two major pathways—the kynurenine pathway and the methoxyindole pathway, in addition to degradation in the intestine. More than 95% of absorbed tryptophan is converted to N- formyl-L-kynurenine and tryptophan 2,3-dioxygenase 2 (TDO) by indoleamine-2,3-dioxygenase (IDO-1 and 2) in liver and the peripheral tissues (**Fig. 1**). The remaining 5% passes through blood brain barrier and is converted to serotonin and melatonin. Tryptophan also undergoes fermentation by intestinal bacteria into indoles, which are absorbed through the portal circulation for further detoxification to indoxyl by the liver microsomal p450 cytochrome system. Indoxyl moieties are then converted to indoxyl sulfate and acetate for excretion from kidneys [2].

Tryptophan metabolites have different physiologic functions depending on the organ system and pathological status. For example, kynurenine serves as a cofactor in the production of nicotinamide adenine dinucleotide (NAD) and also has been implicated in cardiovascular diseases, infections, transplant rejection, allergies, cancer immunity and autoimmunity [3-7]. The methoxyindole pathway generates serotonin (5-hydroxytryptamine), a neurotransmitter and a substrate for melatonin, and both have a predominant function in neurophysiology (**Fig. 1**). While the physiologic function of indole metabolites in human remains unknown, their retention has been implicated in several complications of chronic kidney disease or uremia, such as thrombosis, atherosclerosis, and innate immunity, and, hence, being appropriately termed as uremic toxins [2,8,9].

Given the integral role of tryptophan and its metabolites in several pathological processes, it stands to reason that their estimation in blood will provide important pathogenic insights and can be further developed as a disease biomarker for specific clinical phenotype. Clinical validation of such tryptophan-based biomarker will help risk stratify patients and may also serve as a predictor of a therapeutic intervention. Integration of such measure will help individualization of clinical care and will drive precision to therapeutic intervention. Therefore, the clinical need for accurate estimation of tryptophan metabolites is obvious.

Recent development of chromatography coupled with mass spectrometry technology has provided a powerful tool for separation and quantitation of small metabolites. While there are previous reports related to methods of estimation of tryptophan metabolites levels in cells, monkey serum and human plasma [10-12], they are limited as they either focused on a small subset of tryptophan metabolites, or have not used both positive and negative modes for metabolites detection. Importantly, most of those methods remain to be validated in humans [10-12]. Addressing these limitations, herein, we describe a comprehensive method to estimate a panel of tryptophan metabolites that are retained in patients with chronic kidney disease on dialysis (end stage renal disease; ESRD). Our method includes an isotope dilution technique, and by using both the positive and negative modes of separation, we have enhanced the sensitivity of detection of each metabolite. The methods were further examined in a nested cohort of patients with ESRD compared to a set of well-matched controls.

## METHODS

### Chemicals, reagents and instruments

Tryptophan, xanthurenic acid, kynurenine, indoxyl acetate, indoxyl sulfate, anthranilic acid, indoxyl sulfate-D4, acetonitrile (ACN), methanol, high performance liquid chromatography (HPLC) water, formic acid and ammonium peroxide were obtained from Sigma Aldrich (MO, USA). L-Kynurenine-d6, indoxyl sulfate-d4, Anthranilic acid-N15 and L-Tryptophan-d8 were obtained from Cambridge Isotope Laboratories (MA, USA). Xanthurenic-d4 acid was obtained from Medical Isotopes Inc. (NH, USA). Kynurenic acid-d5 acid was obtained from CND Isotopes Inc. (Quebec, Canada). 3cc HLB cartridge, 30 mg Atlantis T3 C18 3µm 50 × 2.1 mm column were obtained from Waters Corporation (MA, USA). All chemicals and reagents were stored at the recommended temperature and conditions. API 4000 triple quadrupole mass spectrometry equipped with electrospray and Agilent HPLC pump G1312B were used in this study.

### Sample preparation

Brieﬂy, 200 µl of serum samples, quality control (QC) samples and standards were diluted with 500 µl of 0.1% formic acid containing an internal standard. All diluted samples were subjected to solid phase extraction (SPE). The cartridge was conditioned with MeOH and 1% formic acid, and subsequently the sample was loaded onto the cartridge and the cartridge was washed with 1% formic acid. The analytes were eluted with 1% ammonium peroxide in methanol/water (50:50). The eluent was dried under nitrogen. The pellet was reconstituted in 150 µl of 5 mmol/l ammonium acetate solution.

### Preparation of stock solutions and internal standard solution

Seven metabolites were weighted, dissolved in methanol, and diluted to specified concentrations in 5% bovine serum albumin/phosphate buffered saline (BSA/PBS) solution. The concentrations of these metabolites in mixed stock standards were as following: tryptophan (100 µM), kynurenic acid (0.4 µM), kynurenine (4 µM), xanthurenic acid (1 µM), anthranilic acid (0.1 µM), indoxyl sulfate (100 µM), indoxyl acetate (10 µM). The mixed stocks were further diluted in 0.5% BSA/PBS solution for preparation of calibration curves. Internal standards preparation was as follows: tryptophan-d8, kynurine-d6, kynurenic acid-d5, xanthurenicacid-d4, anthranilic acid-N15 and indoxyl sulfate-d4 were dissolved in methanol at a final concentration (20 µg/ml for tryptophan-d8, kynurine-d6, kynurenic acid-d5, xanthurenicacid-d4 and indoxyl sulfate-d4, and 50 µg/ml for anthranilic acid-N15). Mixed internal standards were diluted with 0.1% formic acid at ratio 1:500 before use. Two QC samples were prepared by spiking a certain amount of analytes into charcoal-treated serum. The two QC samples were aliquoted and stored in **−**20°C freezer.

### HPLC and mass spectrometry analysis

Atlantis T3 C18 column was used for the separation of tryptophan metabolites. A binary gradient consisting of solvent A (5 mmol/l ammonium acetate) and solvent B (methanol) was applied. In positive mode, we kept solvent A at 95% for 0.5 min, decreased solvent A to 5% from 0.5 min to 8 min, and then returned solvent A to 95%. In negative mode, solvent A was kept at 95% for 0.5 min, and then solvent A was decreased to 5% from 0.5 min to 5 min, and further decreased to 2% from 5 to 8.5 min, and then was returned to 95%. The flow rate was 0.25 ml/min and the injection volume was 10 µl.

### Mass spectrometry detection

**Figure 2.**
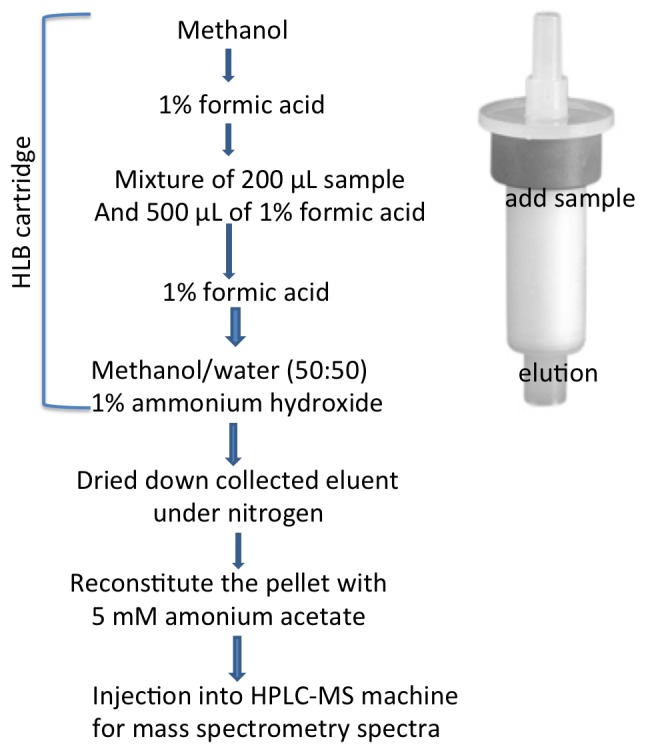
The schematic of sample processing. The above schema shows the processing steps of the serum before injecting into HPLC-MS machine.

Mass spectrometry was equipped with an electrospray source. All seven metabolites and their internal standards were infused into mass spectrometry for scanning spectrum and determining fragmentation. Selected parent/fragment pairs for each uremic solute were used for mass spectrometry detection. The molecules undergo ionization in either of the modes in the mass spectrometry depending on their molecular structures. While developing a quantitative method for any metabolite, it is standard practice to check for its ionization in both the modes, since better ionization enhances recognition and quantification of the metabolite. This exercise is not needed once the method is established. **Table 1** describes the preferable mode of separation of a given metabolite. Tryptophan, kynurenine, kynurenic acid and xanthurenic acid were detected in positive mode, while indoxyl sulfate, indoxyl acetate and anthranilic acid were detected in negative mode. Each metabolite has a stable isotope-labeled metabolite as internal standard. Mass spectrometry condition for positive mode is CAD 6, CUR15, GS1 50, GS2 40, IS 4000, TEM 550, DP 48 and EP 10. Mass spectrometry condition for negative mode is CAD 6, CUR20, GS1 45, GS2 20, IS 4500, TEM 350, DP 48 and EP 10. **Figure 2** shows a schematic of the sample processing. **Table 1** provides the details on metabolite detection.

### Linearity and low limit of quantitation

The linearity in the range of detection of each analyte was analyzed by dilution of individual stock solution with 5% PBS. Each concentration was measured 3 times. Linearity and low limit of quantitation (LLOQ) was also determined by the same way until coefficient variation was large than 15%.

### Intra-assay and inter-assay variation

In order to test the precision of the LC/MS assay, intra-assay variation was conducted by determining the coefficient variation of six aliquots of QC samples. The two QC samples were also analyzed in 3 different days to calculate inter-assay variation.

### Recovery

To evaluate the accuracy of the assay, various amounts of an analyte was spiked to human serum and further processed. The recovery was calculated by the following formula: Percent Recovery = (amount in spiked sample - amount in non-spiked sample)/spiked amount × 100%.

### Human subjects and serum collection

Pre-dialysis blood was collected from patients with end stage renal disease on hemodialysis. Age- gender- and ethnic background-matched subjects served as their controls. Ten ESRD patients on hemodialysis (HD) were randomly recruited from a pool of 150 patients at the DaVita Hemodialysis Center (Boston, MA). The protocol was approved by the Institutional Review Board of Boston University Medical Center. Informed consents were obtained and 10 ml of blood collected prior to the next HD session. Patients with hemoglobin < 8 gm/dl were excluded. Control sera matched for age-, gender- and ethnicity-matched subjects were obtained from Research Blood Component Inc. (Boston, MA). Controls with creatinine more than 1.0 mg/dl were excluded.

### Statistical analysis

Summary statistics are presented as the mean and standard deviation (SD). A paired-*t* test or Wilcoxon rank sum test was performed to compare the groups as appropriate using Graphpad Prism^®^. Statistical significance was assessed at the *P* < 0.05.

## RESULTS

### Method development

**Figure 3.**
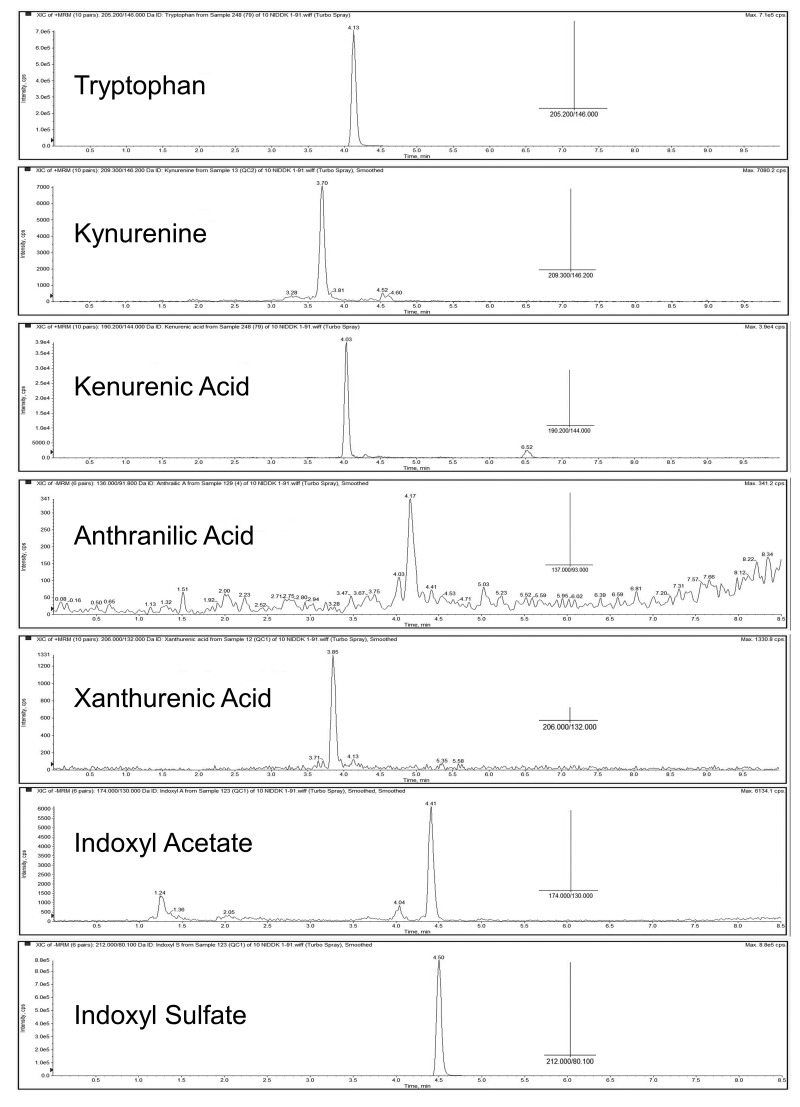
Separation and detection of all metabolites. Separation was on Atlantis T3 C18 3µm 50 × 2.1 mm column Waters Corporation (MA, USA). A binary gradient consisting of solvent A (5 mmol/l ammonium acetate) and solvent B (methanol) and different gradients were applied (please see the method section for specific details about each metabolite). Mass spectrometry of each metabolite was examined using API 4000 triple quadrupole mass spectrometry equipped with electrospray and Agilent HPLC pump G1312B using plasma samples. All seven metabolites and their internal standards were infused into mass spectrometry for scanning spectrum and determining fragmentation. Selected parent/fragment pairs for each uremic solute are shown. The X-axis is time (minutes of elution time of the HPLC column) and Y-axis is intensity of counts per second. The insert is MS/MS data.

After comparing different columns and mobile phases, Atlantis T3 C18 column was chosen, as it provided better separation. As shown in **Figure 3**, tryptophan and its six metabolites were well separated. The selected fragments for identification of the seven products and their mass are presented in **Figure 3**.

### Method linearity

Isotope dilution technique was used for quantitation of tryptophan and its metabolites. The linear ranges for the seven compounds are 0.1 to 500 µM for tryptophan, 0.01 to 10 µM for anthranilic acid, 0.1 to 500 µM for indoxyl sulfate, 0.01 to 10 µM for indoxyl acetate, 0.002 to 20 µM for kynurenic acid, 0.04 to 40 µM for kynurenine and 0.001 to 10 µM for xanthurenic acid, as shown in **Table 2.** Most of the metabolites could be quantitated in lower µM to nM range as shown in **Table 3**.

### Precision and accuracy

The intra-assay and inter-assay of each metabolite in QC1 and QC2 samples were determined by calculating the correlation coefficient (CV). Triplicate runs were performed and SD and CV (%) were calculated to ascertain the precision of the LC-MS analysis, as presented in **Table 4.** All values were in acceptable range, which includes variance within 15% per FDA/society criteria [13]. In intra-assay variation, tryptophan was quantitated with 4.189 CV (%) which was very similar to the inter-assay variation of 6.550. Out of the seven compounds tested, in intra-assay variation kynurenine [CV (%) 10.209] showed the highest variation while xanthurenic acid was quantitated with the lowest [CV (%) 2.741]. Similarly, in inter-assay variation indoxyl acetate showed CV (%) of 13.681 and again xanthurenic acid with the least CV (%) of 3.905 (**Table 4**).

Accuracy was determined by the recovery test. The known quantities of the compounds were spiked to plasma. Recoveries of all the spiked metabolites were in the range of 82%–115% as represented in **Table 5**.

### Biological validation

**Figure 4.**
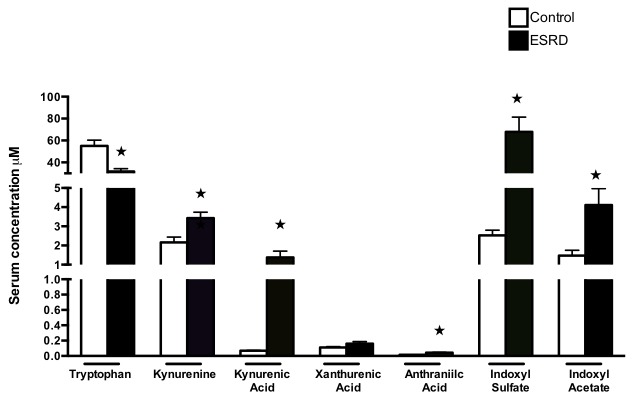
Method validation: significant increases in indoxyl sulfate and indoxyl acetate and kynurenine levels in ESRD patients. All the metabolites were estimated in pre-dialysis sera samples obtained from patients with ESRD and compared to their matched controls (*N* = 10 for both the groups). Shown are averages of sample detection done in duplicates. Tryptophan levels were significantly lower in ESRD patients (*P* = 0.007). Compared to the controls significantly higher levels of kynurenine (*P* = 0.006), kynurenic acid (*P* = 0.0007), anthranilic acid (*P* = 0.002), indoxyl sulfate (*P* = 0.0001) and indoxyl acetate (*P* = 0.0092) were detected. Error bars = SEM.

Having established sensitive and accurate assays of the different tryptophan metabolites, we corroborated the usefulness of these methods in analyzing sera samples from a case-control human cohort. To this end, a set of 10 sera samples from ESRD patients collected before initiation of hemodialysis were compared to age-, gender and ethnic-background matched controls. Baseline characteristics of the current subset of patients are representative of the population treated in our medical center, comprising of 9 African Americans and 1 Hispanic patient, all males, with median age of 43 years (range 27–51 years) and relatively high BMI (median 26.1, range 17–31). Diabetes and hypertension dominated as the cause of end stage renal disease. The levels of indoxyl sulfate, indoxyl acetate and kynurenine, kynurenic acid and anthranilic acid were significantly elevated in the ESRD cohort, compared to control samples (**Fig. 4**), while xanthurenic acid levels remained unchanged between them. Higher levels of indoxyl sulfate, indoxyl acetate and kynurenine, as expected in ESRD patients validate the above described methods [8,10].

## DISCUSSION

The current study describes a comprehensive method of precisely and collectively determining total levels of tryptophan and its metabolites, as duly validated in a group of ESRD patients. Human serum is a complex mixture, consisting of a multitude of soluble proteins, lipids, metabolites, *etc.*, making the detection of small metabolites quite challenging. LC/MS represents an ideal technique to quantify the metabolites. Although several methods have been published [10-12,14,15], we developed sensitive methods for estimating the level of a panel of tryptophan metabolites in human sera, some of which were not previously determined.

Zhu *et al*. have described a method for analyzing tryptophan, kynurenine, kynurenic acid and xanthurenic acid [14]. While their LLOQ values were similar to our values (**Table 3**), the validation in humans was not performed for their methods. Recently, Fuertig *et al*. used LC-MS/MS method to measure tryptophan metabolites in monkey plasma and cerebrospinal fluid [15]. Though the sensitivity of their method was better than those described here, their LLOQ determination was performed with matrix-free calibration standard, which can explain their better sensitivity. However, such method is likely to meet difficulties in heterogeneous samples such as human serum. Compared to the above methods, in our study, both positive mode and negative modes were applied, enhancing the sensitivity of detection of each metabolite, as it varies in different modes. Tryptophan, kynurenine, kynurenic acid and Xanthurenic acid showed higher detection in positive mode, while indoxyl sulfate, indoxyl acetate and anthranilic acid exhibited better detection in negative mode. The methods were validated in human samples. Thus, the methodology described here is sensitive with higher accuracy of estimating metabolites optimized for each tryptophan metabolite with different characteristics. Our inter-assay, intra-assay variation, recovery and linearity are in FDA acceptable range [13], underscoring the precision and translational research applicability of these tests.

Albumin is a predominant protein in serum and serves as a vehicle for several uremic solutes in blood such as indoxyl sulfate and indoxyl acetate [2,9]. Almost 90% of indoxyl sulfate and indoxyl acetate circulate as albumin-bound and only 10% remains free in blood. While the free forms of these metabolites are considered pathogenic, and other methods of measuring specifically the albumin-bound and the free forms of these metabolites have been described [16-18], the current methods estimate the total solutes in blood. However, this limitation is not clinically relevant, as it is possible to extrapolate the free fraction of each solute with this method, given the percentage of albumin-bound (10%) fraction is known for each metabolite and both the fractions exist in equilibrium with each other.

Our intention of selecting pre-dialysis blood for estimating metabolite levels was driven by several factors. First, this strategy integrates well in the clinical flow of the dialysis unit, as most of blood collections for ESRD patients are done before the initiation of dialysis. Thus, this strategy avoids a separate venipuncture of the patient for research purpose. Second, our intention was to avoid any variations in metabolite concentrations with hemodialysis procedure, even though highly protein-bound metabolites, such as indoxyl sulfate and indoxyl acetate are poorly dialyzed and their levels are unlikely to change with the dialysis. However, this is not the case with tryptophan, which up to 90% circulates in blood in an unbound form and may get dialyzed out. Interestingly, our data showed a significantly lower level of tryptophan in pre-dialysis samples in ESRD patients compared to controls (**Fig. 4**), which may be due to dietary deficiency in line with the generalized malnutrition in ESRD patients along with the cumulative loss of free tryptophan in dialysis [19].

These assays are developed as part of a core facility at Boston University School of Medicine to allow its wide application to several investigators in their respective areas of research. While the quality matrices of these methods are in congruence with the FDA standards [13], allowing their use in translational research, we believe that further quality control of these tests (*e.g.*, via analysis of various other cohorts) can make them suitable for Clinical Laboratory Improvement Amendment (CLIA) certification and allow their use for clinical purposes. In conclusion, we report here sensitive LC/MS-based methods to accurately quantify a comprehensive panel of tryptophan metabolites, the “tryptophanome”. These methods bring us closer to individualizing the risk stratification for patients with renal disease, and could add precision to their clinical management.

## Figures and Tables

**Table 1.** Detection and quantification of the metabolites by mass spectrometry.

Metabolites	Mode	MW	Q1 (M/Z)	Q3 (M/Z)	CE (volt)	Internal standard
Tryptophan	+	204.2	205.2	146.0	25	Tryptophan-d8
Kynurenine	+	208.2	209.3	146.0	26	Kynurine-d6
Kynurenic acid	+	189.2	190.2	144.0	30	Kynurenic acid-d5
Xanthurenic acid	+	205.2	206.0	132.0	42	Xanthurenicacid-d4
Indoxyl sulfate	−	213.2	212.0	80.1	−35	Indoxyl sulfate-d4
Indoxyl acetate	−	175.2	174.0	130.0	−15	Indoxyl sulfate-d4
Anthranilic acid	−	137.2	136.0	91.8	−32	Anthranilic acid-N15

CE: collision energy; MW: molecular weight (Kda); Q (M/Z): mass/charge ions. +: positive separation; -: negative separation

**Table 2.** Linearity of detection.

µM	Tryptophan	Kynurenic acid	Kynurenine	Xanthurenic acid	Anthranilic acid	Indoxyl sulfate	Indoxyl acetate
Linearity range	0.1–500	0.002–20	0.04–40	0.001–10	0.01–10	0.1–500	0.01–10
*R^2^ value*	0.9989	0.9991	0.9998	0.9995	0.9993	0.9991	0.9973

*R*^2^ = correlation coefficient.

**Table 3.** Lowest quantitation limit of tryptophan metabolites by LC-MS method.

Quantitation limit (µM)	Tryptophan	Kynurenic acid	Kynurenine	Xanthurenic acid	Anthranilic acid	Indoxyl sulfate	Indoxyl acetate
	0.010	0.001	0.016	0.005	0.013	0.100	0.005

**Table 4.** Inter assay and intra-assay variability of estimation of uremic solutes.

	Tryptophan	Kynurenic acid	Kynurenine	Xanthurenic acid	Anthranilic acid	Indoxyl sulfate	Indoxyl acetate
Intra-assay variation							
QC1							
Average (µM)	7.471	0.037	0.687	0.146	0.033	16.003	0.127
SD	0.197	0.001	0.086	0.004	0.003	0.377	0.012
CV (%)	2.633	2.524	12.511	2.644	9.143	2.357	9.750
QC2							
Average (µM)	22.968	0.096	1.510	0.436	0.074	46.044	0.441
SD	0.962	0.004	0.154	0.012	0.005	1.838	0.034
CV (%)	4.189	4.007	10.209	2.741	6.137	3.991	7.800
Inter-assay variation							
QC1							
Average	7.951	0.034	0.763	0.150	0.032	16.065	0.136
SD	0.740	0.003	0.067	0.003	0.004	0.065	0.013
CV (%)	9.309	7.796	8.819	2.019	13.548	0.403	9.446
QC2							
Average	23.870	0.097	1.573	0.447	0.066	44.578	0.421
SD	1.563	0.001	0.128	0.017	0.008	2.606	0.058
CV (%)	6.550	0.978	8.121	3.905	11.938	5.847	13.681

**Table 5.** Recovery of uremic solutes.

	Tryptophan	Kynurenic acid	Kynurenine	Xanthurenic acid	Anthranilic acid	Indoxyl sulfate	Indoxyl acetate
Add (µmol)	16.670	0.067	1.333	0.333	0.033	33.300	0.333
Recovery (%)	112.440	111.672	93.060	96.696	99.568	108.724	90.296
SD	6.674	7.269	4.603	2.598	3.330	3.395	8.008
CV (%)	5.936	6.509	4.946	2.687	3.344	3.122	8.869
							
Add (µmol)	5.000	0.020	0.400	0.100	0.010	10.000	0.100
Recovery (%)	108.1	107.396	82.457	96.042	105.615	115.321	90.000
CV (%)	5.105	7.870	8.028	2.835	4.481	2.691	5.214
